# Working memory load impairs tacit coordination but not inter-brain EEG synchronization

**DOI:** 10.1093/scan/nsae017

**Published:** 2024-02-28

**Authors:** Lionel A Newman, Ming Cao, Susanne Täuber, Marieke van Vugt

**Affiliations:** Engineering and Technology Institute, University of Groningen, Groningen 9747 AG, The Netherlands; Bernoulli Institute for Mathematics, Computer Science and Artificial Intelligence, University of Groningen, Groningen 9747 AG, The Netherlands; Engineering and Technology Institute, University of Groningen, Groningen 9747 AG, The Netherlands; Department of Human Resource Management & Organizational Behavior, University of Groningen, Groningen 9747 AG, The Netherlands; Bernoulli Institute for Mathematics, Computer Science and Artificial Intelligence, University of Groningen, Groningen 9747 AG, The Netherlands

**Keywords:** theory of mind, joint action, working memory, hyperscanning, synchronization

## Abstract

Coordinating actions with others is thought to require Theory of Mind (ToM): the ability to take perspective by attributing underlying intentions and beliefs to observed behavior. However, researchers have yet to establish a causal role for specific cognitive processes in coordinated action. Since working memory load impairs ToM in single-participant paradigms, we tested whether load manipulation affects two-person coordination. We used EEG to measure P3, an assessment of working memory encoding, as well as inter-brain synchronization (IBS), which is thought to capture mutual adjustment of behavior and mental states during coordinated action. In a computerized coordination task, dyads were presented with novel abstract images and tried selecting the same image, with selections shown at the end of each trial. High working memory load was implemented by a concurrent n-back task. Compared with a low-load control condition, high load significantly diminished coordination performance and P3 amplitude. A significant relationship between P3 and performance was found. Load did not affect IBS, nor did IBS affect performance. These findings suggest a causal role for working memory in two-person coordination, adding to a growing body of evidence challenging earlier claims that social alignment is domain-specific and does not require executive control in adults.

Humans are able to coordinate actions with other individuals, even without explicit communication about intentions or beliefs, such as when dancing, merging into traffic or deciding where to meet with a new acquaintance. Until recently, the neural mechanisms that allow us to perform such tacit coordination have almost exclusively been studied in single-participant paradigms where participants view images of social actions and answer questions about implicit intentions and beliefs of onscreen characters. Lately, a shift towards studying more dynamic interaction has led some researchers to investigate behavior in two-person coordination tasks derived from game theory, while at the same time utilizing novel measures of brain-to-brain coupling thought to capture mutual perspective-taking. However, researchers have yet to firmly establish a causal role for any specific cognitive mechanisms in tacit coordination, and the extent to which recent brain-to-brain measures reflect such mechanisms remains a matter of debate. Our study aims to provide insights into these questions.

In game theory, tacit coordination can be formalized in pure coordination games in which players receive maximal benefits when selecting the same response. To optimize performance, players must use what Schelling (1960) classically termed a ‘focal point’—a salient source of information known to each player. For example, in Mehta *et al*. (1994), participants were asked to either give the same name as a random partner (coordination condition) or give any name (control condition). The name ‘John’ was given by 50% of participants in the coordination condition, but only 9.1% in the control condition. In the coordination condition, participants used a focal point: the shared belief that ‘John’ was a common name likely to be chosen by a partner. A large body of evidence suggests that using focal points requires theory of mind (ToM)—the ability to attribute underlying mental states, such as intentions and beliefs—as well as hierarchical abstraction in order to build higher-order ToM representations (e.g. player A’s prediction about how player B thinks that player A will respond) (Yoshida *et al*., 2008; Bardsley *et al*., 2010; McMillan *et al*., 2012; de Weerd *et al*., 2015; Xie *et al*., 2020).

Such ToM processes likely require domain-general cognitive resources such as working memory. Evidence for a causal role of working memory comes from dual-task experiments manipulating working memory load during ToM tasks. ToM performance varies as a function of working memory load imposed by a concurrent task, implying that greater demand for working memory by concurrent tasks leaves fewer resources available to allocate towards ToM processing. These single-participant experiments suggest that working memory plays an important role in the selection between processing one’s own perspective or, alternatively, the other’s perspective (McKinnon and Moscovitch, 2007; Bull *et al*., 2008; Qureshi *et al*., 2010; Maehara and Saito, 2011; Qureshi and Monk, 2018).

It remains unclear to what extent these findings about working memory from single-participant experiments generalize to more naturalistic situations in which two or more individuals interact (Sebanz *et al*., 2006; Konvalinka and Roepstorff, 2012; Schilbach *et al*., 2013). So far, only a small number of studies have explored ‘correlational’ relationships between working memory measures and pure coordination (Mizrahi *et al*., 2020), and no studies to our knowledge have investigated a more ‘causal’ role for working memory by experimentally manipulating working memory during pure coordination.

When investigating the effects of working memory manipulation on coordination, concomitant effects on brain measures may also offer insights into mechanisms for coordination. A particular type of brain measure that is of significant interest in studying multi-participant tasks is hyperscanning: the simultaneous recording of brain activity from two or more participants during interaction. The goal of hyperscanning is to capture interpersonal neural dynamics involved in alignment of goals and actions (Sebanz *et al*., 2006; Becchio *et al*., 2010; Konvalinka and Roepstorff, 2012; Schilbach *et al*., 2013; Keller *et al*., 2014). Importantly, hyperscanning allows researchers to measure (relationships between) multiple participants’ brain activity in real time as focal points emerge during coordination.

Building on initial findings from Montague *et al*. (2002), a growing number of hyperscanning studies using electroencephalogram (EEG), magnetoencephalogram (MEG), functional magnetic resonance imaging and function near-infrared spectroscopy have reported increased inter-brain synchronization (IBS) in socially interactive conditions (for reviews, see Mu *et al*., 2018; Wang *et al*., 2018; Czeszumski *et al*., 2020). Increases in IBS are frequently reported in brain regions previously implicated in ToM, notably in the temporoparietal junction (Tang *et al*., 2016; Jahng *et al*., 2017; Gvirts and Perlmutter, 2020; Xie *et al*., 2020), anterior cingulate cortex (Babiloni *et al*., 2007; De Vico Fallani *et al*., 2010; Astolfi *et al*., 2010b) and inferior frontal gyrus (Saito *et al*., 2010; Yun *et al*., 2012; Osaka *et al*., 2015). Similarly, in EEG and MEG, these increases are typically in frontal and temporoparietal regions and in the alpha and theta frequency band (Lachat *et al*., 2012; Sänger *et al*., 2012; Kawasaki *et al*., 2013; Toppi *et al*., 2015; Jahng *et al*., 2017; Ahn *et al*., 2018; Hu *et al*., 2018).

These findings suggest that IBS may reflect mutual perspective-taking (Dumas *et al*., 2011; Riley *et al*., 2011; Hasson *et al*., 2012; Czeszumski *et al*., 2020). To our knowledge, no studies have yet tested this interpretation explicitly by investigating the relationship between IBS and performance in pure coordination games. Nor are we aware of previous research investigating whether IBS is sensitive to manipulation of domain-general processes that influence ToM, such as working memory.

While IBS in pure coordination games remains largely unexplored, IBS has been measured in dominance-solvable coordination games like the Prisoner’s Dilemma (De Vico Fallani *et al*., 2010; Astolfi *et al*., 2010a, 2011; Jahng *et al*., 2017; Hu *et al*., 2018). However, we believe that interpretation of IBS in dominance-solvable scenarios is problematic. While players in both pure coordination and dominance-solvable games use ToM to predict each other’s actions, those in dominance-solvable games can then use predictions to either cooperate or exploitatively maximize their own benefit at the expense of the other player. Therefore, behavior in dominance-solvable games depends on additional processes beyond ToM alone (Kuo *et al*., 2009; Duffy and Smith, 2014; Bear and Rand, 2016). Contrasts between cooperation and exploitation may not be ideal for tracking the perspective-taking processes that IBS is theorized to reflect, since these processes are important for both cooperation and exploitation (Gallotti *et al*., 2017). This may explain mixed findings in which higher IBS has sometimes been associated with cooperation (De Vico Fallani *et al*., 2010; Hu *et al*., 2018) and sometimes with exploitation (Astolfi *et al*., 2010a; Jahng *et al*., 2017; Ciaramidaro *et al*., 2018). Therefore, rather than investigating IBS in terms of contrasts between ‘cooperation or exploitation’, it may be more productive to explore differences in IBS dependent on ‘accuracy of predictions for behavior’ during coordinated action. Pure coordination games allow this exploration, and are therefore likely to be useful in determining whether IBS is sensitive to ToM processing and its contributing domain-general resources.

To that end, we conducted a dual-task hyperscanning EEG study in which participants engaged in a pure coordination task together with a concurrent working memory task. First, to expand on traditional single-participant ToM studies, we hypothesized that working memory load impairs two-person coordination performance. Second, to investigate a widely held but untested interpretation of IBS, we hypothesized that IBS in frontal alpha oscillations—one of the most frequently observed EEG IBS signatures—varies as a function of load.

## Methods

### Participants

Eighty-six participants aged 18–35 years old (mean = 23.14, SD = 4.06) took part in the study. Participants were randomly matched into 43 same-sex dyads (9 male–male pairs, 34 female–female pairs), since IBS differs between same-sex and mixed-sex dyads (Cheng *et al*., 2015; Baker *et al*., 2016). Technical failure resulted in loss of behavioral data for one dyad and loss of EEG data for three additional dyads. All participants were right-handed, reported having normal or corrected-to-normal vision, passed a color blindness test and reported having no history of neurological injury or illness.

To prevent the effects on IBS and coordination that have been previously associated with intimate relationship (Kinreich *et al*., 2017; Goldstein *et al*., 2018; Reindl *et al*., 2018), we ensured dyads were not friends, romantic partners or family. Upon arrival to the location of the study, participants were introduced to each other and were able to chat during experimental setup.

In total, participants took approximately 2 h to complete the experiment: approximately 15 min for questionnaires (see Supplementary Material), 45 min for EEG setup and 1 h for the behavioral task. The experiment and recruitment took place at the University of Groningen. All procedures were approved by the Research Ethics Committee (CETO). All participants provided written informed consent and received monetary compensation.

### Task

Dyads performed a computerized pure coordination task in the same room while seated at separate tables, each with its own monitor (set at identical resolution) and separate keyboard. Visual contact was blocked by a floor-to-ceiling bookcase, and participants were instructed not to communicate verbally during the task, which was monitored by experimenters.

**Fig. 1.**
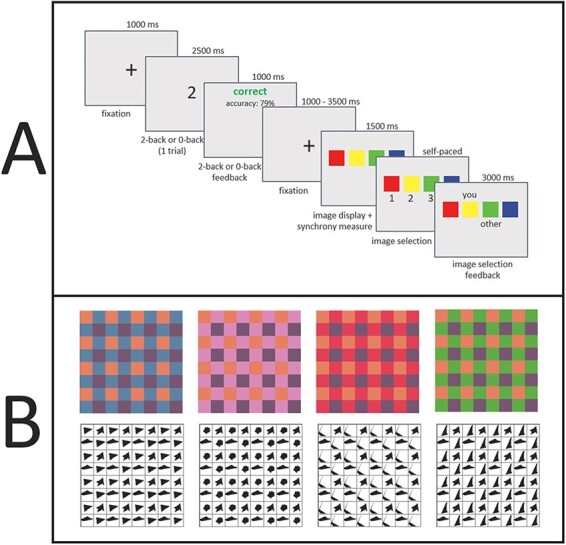
Trial sequence (A) and example stimuli (B). Following a 1000 ms fixation cross, a number was displayed for 2.5 s while participants responded by key press whether the number was even or odd (low load block) or whether the number was the same as or different from the number shown two trials ago (high load block). After this single trial of the number task, feedback was shown for 1 s, followed by a jittered fixation cross (1–3 s, randomized for each dyad in each trial) to prevent EEG synchronization resulting solely from the predictability of stimulus onset timing. Then, participants viewed four novel images (shapes in one block, colors in the other block) and, after 1.5 s, were able to select an image. After both participants made their selections, all images remained onscreen while the chosen images were labeled with ‘you’ for the participant’s own choice and ‘other’ for the other participant’s choice. After displaying this feedback for 3 s, a new trial began.

At the beginning of each block, participants completed practice trials (see Supplementary Material). The task consisted of two blocks, 90 trials each, separated by a self-paced break. Trial sequence is shown in Figure 1. Stimuli were adapted from Alberti *et al*. (2012) (see Supplementary Materials). In each trial, participants viewed four novel images composed of alternating shapes or colors, and were instructed to select the same image as their partner. The images displayed in each trial were arranged on a horizontal plane, with left-to-right order randomized separately for each participant. Participants were informed of this so that their selections had to be based on image features rather than positions. Participants could make an image selection starting 1.5 s after image onset which allowed us to capture event-related potentials (ERPs) that were not contaminated with response-making. While images were displayed, participants made two self-paced responses, a ‘first-best guess’ and ‘second-best guess’ (only first-best guesses were used in this report). Participants responded using a right-hand key press. At the end of each trial, the images selected as first-best guess by both participants were displayed for 3 s, allowing participants to become familiar with their partner’s choices. Participants typically converge on shared decision rules by each forming a belief about the other’s preferences based on which image features have been chosen in previous trials, and adjusting their subsequent choices accordingly. For example, based on their previous selections, both participants might eventually decide to select the darkest image, the image with the most red or the image containing a certain shape which was present in previous selections. Thus, this pure coordination task serves as a simplified but empirically tractable method for operationalizing multi-agent interactions where each individual’s previous actions mutually influence their subsequent actions, ideally towards alignment of intentions, beliefs and behaviors.

To manipulate working memory, we interleaved a different task. Before the image coordination phase of each trial, a fixation cross was displayed for 1 s, followed by a number. In the low load block, participants reported by button press whether the number was even or odd. In the high load block, participants performed a standard two-back task (Kirchner, 1958), in which they reported whether the displayed number was the same or different from the number shown two trials ago (see Supplementary Material), thus requiring participants to maintain numbers in working memory during the decision-making portion of the task.

To prevent emergent decision rules from carrying over between high load and low load blocks, one block used color images only while the other block used shape images only. This resulted in a 2 (color *vs* shape stimuli) by 2 (high *vs* low load) within-subjects design. The block conditions and their order were counterbalanced across dyads.

Participants were compensated with a base rate of €8 per hour, plus a bonus of up to €4 more depending equally on both image coordination and n-back performance to minimize the likelihood that participants would bypass the working memory load manipulation by focusing solely on image selection while neglecting the n-back (see Supplementary Materials).

### EEG recording and pre-processing

Two separate 32-channel systems (BioSemi) were used to record EEG with a sampling rate of 512 Hz. The two systems recorded simultaneously using the BioSemi ActiveTwo daisychain mode (see Figure 8 in Barraza *et al*., 2019). More details are provided in the Supplementary Materials.

### Data analysis

#### Behavioral analyses

##### Interaction-specific effects on performance.

Following previous investigations of focal point coordination, ‘matching frequency’ and ‘coordination index’ were calculated as summary statistics for performance (Mehta *et al*., 1994; Bardsley *et al*., 2010; Alberti *et al*., 2012; McMillan *et al*., 2012). Matching frequency is the proportion of correctly matched choices between paired, interacting participants. Coordination index is the probability that two non-interacting participants, chosen at random from the entire sample, select matching responses on a given set of four images (that is, a given trial). A matching frequency greater than the coordination index indicates a positive contribution of dyad-specific interaction to performance.

To compute the coordination index, a sample distribution was generated by a permutation test. Specifically, 1000 samples were generated by randomly selecting with replacement the choices of two non-interacting participants on each image set. The distribution of mean correct in these generated samples was compared against the empirical matching frequency. A matching frequency greater or equal to the 0.95 quantile of coordination index indicates matching frequency significantly exceeds the coordination index (α = 0.05).

##### Effects of load on mean performance.

To determine whether mean dyad coordination performance was significantly affected by load, a linear mixed-effects model (LMM; model 1, see Supplementary Table S1) was fitted using maximum likelihood. Fit of each model was compared, by chi-square test and Akaike information criterion (AIC), to a reduced model which included identical terms but excluded load condition as a fixed effect. For all LMM analyses below, we used this model selection procedure where the fit and AIC of a full LMM is compared with a reduced LMM excluding the variable of interest (e.g. load condition). For details, see Supplementary Materials.

##### Effects of load on non-linear changes in performance time-series.

In addition to analyzing the effect of load condition on mean dyad performance, we also analyzed its effect on change in dyad performance across trials. We did this using Generalized Additive Models (GAMs) in R (‘gam’ function in the ‘mgcv’ package) (Wood, 2006). The GAM is a flexible technique that is able to capture both linear and non-linear relationships (for details, see Supplementary Materials; for a tutorial, see van Rij *et al*., 2019). We opted to use this method because, as is common in time-series data, changes in performance across trials—the learning process that participants undergo in converging towards shared choices—were not linear.

Using a logistic GAM, we tested whether load condition had a significant effect on change in accuracy over trials. We fitted a model (model 2) using stimulus type and load condition as parametric (non-spline) predictors, and we implemented the non-linear interactions that account for change in accuracy over trials using ‘smooth’ terms. A one-dimensional non-linear regression line is fitted for smooth terms for each level of the specified conditions. Three smooth terms were estimated in our GAM: (i) the interaction between trial and stimulus type (fitting a non-linear relationship between trial and accuracy for each stimulus type); (ii) the interaction between trial and load condition (fitting a non-linear relationship between trial and accuracy for each load condition); and (iii) a random factor smooth to account for variation between dyads (analogous to random slope in standard LMMs).

#### EEG analyses

##### Event-related potentials.

To assess whether working memory load manipulation affected electrophysiological markers of working memory processing, we measured the amplitude of the P3 ERP component time-locked to stimulus onset in the image coordination task. For background about ERPs, see Supplementary Materials. A lower P3 amplitude in the high load compared with low load condition would indicate allocation of fewer resources for processing of images during coordination.

P3 was calculated with a 1000 ms pre-stimulus baseline. To determine the time window for the P3, ERPs were visually inspected by plotting the average of all conditions to avoid biasing the time window in a way that would reflect differences between high and low load (Luck and Gaspelin, 2017), which resulted in a 400–600 ms post-stimulus window. To determine the effect of load on P3, for each participant, the average amplitude was calculated over the time window in each of the four conditions (color stimuli-low load; color-high; shape-low; shape-high). One participant’s P3 was found to be more than four standard deviations from the mean and was excluded from ERP analyses. For the remaining 77 participants, we fitted a LMM testing the effect of load on P3 amplitude (model 3).

##### Inter-brain synchronization measurement.

IBS was measured for each dyad in the 3-s feedback period at the end of each trial. IBS was calculated separately for delta (0.5–4 Hz), theta (4–7 Hz), alpha (9–14 Hz), beta (12–35 Hz) and gamma (35–50 Hz) frequency bands using Circular Correlation (Ccorr), which has been shown in simulations to be less susceptible to spurious synchronization than common measures like phase-locking value (Burgess, 2013). We computed IBS for all possible homologous electrode pairs, and threshold for statistical significance was adjusted using the False Discovery Rate (FDR) procedure to correct for multiple comparisons across electrodes within each frequency band (Durka *et al*., 2004).

##### Relationships between mean IBS, load, and mean performance.

We then performed two tests on mean IBS: one to examine whether IBS differed between high and low load conditions (model 5), and another to test the effect of IBS on coordination performance (model 6).

## Results

### Behavioral results

#### Interaction-specific effects on performance

To test whether coordination performance (i.e. within-dyad matching responses) was driven by dyad-specific interaction or by task stimuli, a baseline coordination index was calculated by permutation test. Matching frequency for both color stimuli (0.72) and shape stimuli (0.57) lay above the 0.95 quantile of the coordination indices (color: 0.41; shape: 0.50), indicating that performance was driven by interaction between paired participants rather than stimuli themselves.

#### Effects of load on mean performance

**Fig. 2.**
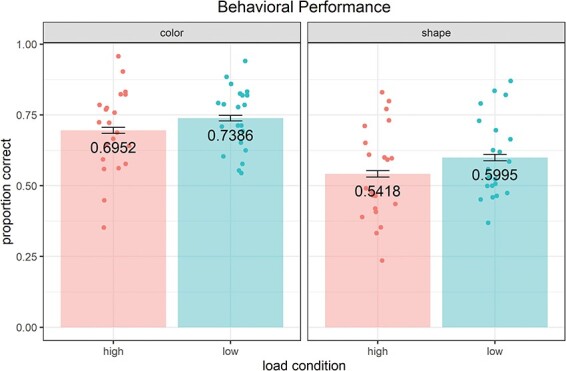
Mean accuracy by load condition and stimulus type. Raw means are shown, with points representing dyads. Error bars represent standard error of the mean. Significant effects on coordination performance were found for both load condition and stimulus type (see Supplementary Table S2).

After having determined that performance was above chance level, we asked whether performance was sensitive to load. Figure 2 shows the mean accuracy for each condition separately. LMM results are shown in Supplementary Table S2. Using a chi-square test to compare the fit of a full LMM (model 1) with a reduced LMM that excluded load condition as a fixed effect, we found that, as predicted, load significantly affected mean accuracy (χ^2^(1)= 3.90, *P* = 0.048), with low load increasing the proportion correct by 0.051 ± 0.025 (standard error). AIC confirmed a preference for the full model (ΔAIC = 1.90). We also found a highly significant effect of stimulus type on mean accuracy by comparing the reduced model with a random-intercept only model (χ^2^(1) = 23.34, *P* < 0.001; ΔAIC = 21.34), with shape stimuli decreasing accuracy by 0.146 ± 0.025 (standard error) compared to color stimuli.

#### Effects of load on non-linear changes in performance Time-series

**Fig. 3.**
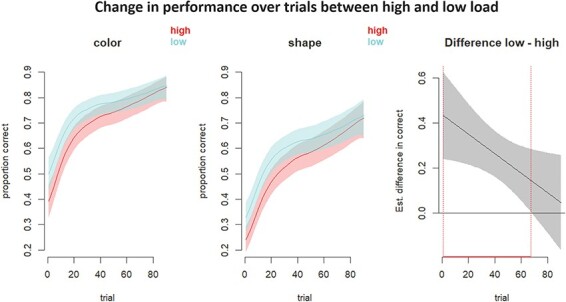
Significant effect of load condition on change in performance over trials. Left and middle panels show estimated effects of high and low load on change in accuracy (left: color stimuli only, middle: shape stimuli only). Right panel shows estimated difference in accuracy collapsed over stimulus type. Shading represents pointwise 95% confidence intervals. GAM analysis revealed a significant effect of load condition on change in accuracy over trials (F = 4.71, edf = 1.004, *P* = 0.031; see Supplementary Table S2). ‘edf’ = estimated degrees of freedom, which is calculated using the number of estimated smoothing parameters, number of parametric (non-smooth) terms and the sum of the penalty null space dimensions for each smooth object (Wood, 2006; Wood, 2017).

Using a GAM (model 2), we found that, as predicted, load condition significantly affected accuracy over trials, as represented by a significant interaction between load condition and trial (F = 4.71, edf = 1.004, *P* = 0.031). Model summary statistics are shown in Supplementary Table S3. To interpret these summary statistics in terms of the extent of the difference between high and low load, and where the curves differ in the trial course, visualization is necessary (van Rij *et al*., 2019). Figure 3 shows that, in the first approximately two-thirds of trials, performance curves remained significantly higher in low load compared with high load for both color and shape stimuli.

### EEG results

#### Event-related potentials

To determine whether our working memory manipulation was successful, we examined an electrophysiological marker of working memory processing, namely the P3 amplitude pooled at 300–500 ms after stimulus onset in the image coordination task. A chi-square test found a superior fit for the full model (model 3) which included working memory load condition as a fixed effect (χ^2^(1)= 7.08, *P* < 0.01) compared to a reduced model excluding load condition. This indicated that, as predicted, load significantly affected P3 amplitude, with low load being associated with an increase in P3 by 0.84 μV ± 0.31 (standard error). AIC confirmed a preference for the full model (ΔAIC = 5.08).

**Fig. 4.**
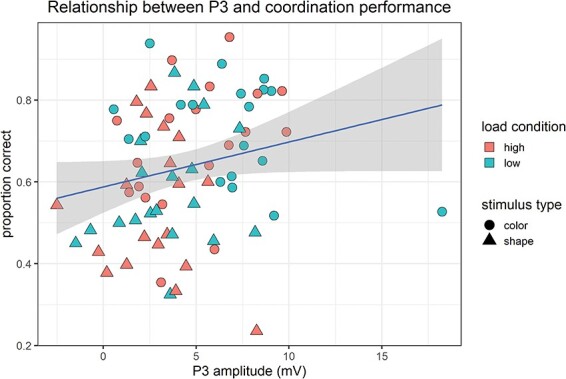
Significant effect of P3 amplitude on performance. Points represent mean accuracy and mean P3 amplitude for each dyad in each condition (two points per dyad, one for each block, i.e. condition). Blue line indicates regression line; gray shaded area indicates 95% confidence intervals.

We then performed a post-hoc analysis to check whether P3 amplitude also affected coordination performance. Consistent with previous analyses, we fitted a LMM for mean accuracy for each dyad in each condition, with dyad-level mean P3 amplitude as a fixed effect and with a random intercept for dyad (model 4). P3 amplitude was found to have a significant effect on coordination performance (see Figure 4), as revealed by a chi-square test comparing the full model to a random-intercept only model (χ^2^(1) = 6.29, *P* = 0.012) and confirmed by AIC (ΔAIC = 4.29).

#### Relationships between Mean IBS, working memory load and mean performance

First, we tested whether mean dyad IBS in any frequency bands differed between high and low load conditions. We did this using chi-square tests and AIC to compare the fit of a full LMM for IBS (model 5) with a reduced model excluding load condition as a fixed effect. No results were found to be significant after repeating this procedure for every homologous electrode pair and correcting *P*-values for multiple comparisons within each frequency band using FDR. Given the surprising lack of findings across all 160 analyses (32 electrodes * 5 frequency bands), we checked for the relative strength of evidence in favor of the null hypothesis (i.e. no effect of load on IBS) over the alternative hypothesis using Bayes Factor analyses (Andraszewicz *et al*., 2015). Specifically, we computed Bayes Factor for each comparison between the full and reduced mixed models using the default priors of the ‘lmBF’ function from the ‘BayesFactor’ package in R. From the 160 analyses, 149 resulted in BF >1, indicating evidence in favor of an absence of the effect of load. The maximum was BF = 4.43 (moderate evidence in favor of an absence of the effect of load), minimum was BF = 0.105 (moderate evidence in favor of an effect of load) and mean was 3.13 (SD = 1.13). The 11 models indicating evidence in favor of an effect of load reflect a range of different frequencies and electrodes: 5 models resulted in BF >0.33 (anecdotal evidence in favor of an effect of load) occurring in alpha (CP2), gamma (O1, F4) and beta (AF4, PO3); and 6 models resulted in 0.33 > BF > 0.10 (moderate evidence in favor of an effect of load) occurring in theta (AF4), alpha (FC2, Fz), gamma (F8, Oz) and delta (O1). A full table including all 160 results is available at the link provided in the Data Availability section.

Next, we used the same procedures to test whether mean dyad IBS contributed to mean dyad accuracy by comparing a full and reduced model (model 6). Again, no results survived correction for multiple comparisons. Following the same Bayes Factor analysis as above, we found that 151 out of 160 analyses resulted in BF >1, indicating evidence in favor of an absence of the effect of IBS on accuracy. The maximum was BF = 4.20 (moderate evidence in favor of an absence of the effect of IBS), minimum was BF = 0.077 (strong evidence in favor of an effect of IBS) and mean was 2.95 (SD = 1.05). In the nine models indicating evidence in favor of an effect of IBS on accuracy, seven resulted in BF >0.33 (anecdotal evidence in favor of an effect of IBS) occurring in gamma (F3, C4, P7), beta (Cz, P3), theta (Fp1, P7); one model resulted in 0.33 > BF > 0.10 (moderate evidence in favor of an effect of IBS) in beta (PO4) and one model resulted in 0.10 > BF > 0.033 (strong evidence in favor of an effect of IBS) in gamma (P3). For this final model, Supplementary Figure 5 shows the relationship between gamma IBS and coordination performance at the P3 electrode. Again, all 160 results are provided in the Data Availability section.

## Discussion

Until recently, interactions between two or more individuals have rarely been investigated in studies measuring brain activity. Our study contributes to the recent shift by social neuroscientists towards a two-brain approach (Schilbach, 2010; Konvalinka and Roepstorff, 2012). Specifically, we expanded on previous single-participant studies that found a causal role for working memory in ToM (McKinnon and Moscovitch, 2007; Bull *et al*., 2008; Qureshi *et al*., 2010; Maehara and Saito, 2011; Qureshi and Monk, 2018) by testing whether working memory load influences the emergence of focal points and IBS during two-person pure coordination. We found that load impairs coordination performance, but it does not affect IBS. The effect of load, along with the effect of P3, on performance in the pure coordination task demonstrate that domain-general cognitive resources play an important role—not only in processing static, pre-given social information as in previous single-participant studies, but also in more naturalistic, dynamic interactions where each individual’s previous actions mutually influence subsequent actions. This adds to the growing body of work challenging earlier claims that social alignment is domain-specific and does not require executive control in adults (Fodor, 1992; Leslie, 1994; Perner and Lang, 1999; Fiddick *et al*., 2000).

Surprisingly, we found no evidence for a role of working memory in IBS. In addition, we found no effect of IBS on coordination performance (although it is worth mentioning the conflicting evidence we found for a possible effect of gamma IBS on performance in the left parietal region). Our lack of clear findings contradicted our expectations informed by influential theories that IBS reflects mutual perspective-taking (Dumas *et al*., 2011; Riley *et al*., 2011; Hasson *et al*., 2012; Czeszumski *et al*., 2020). Taken alone, our findings seem to suggest that IBS does not reflect ToM and its contributing domain-general processes, at least in turn-based interactions where individuals are unable to continuously track and respond to each other’s actions in real-time. However, this stands in contrast to previous hyperscanning studies indicating that observing action outcomes at the end of each turn is sufficient to induce changes in both coordination performance and IBS (Liu and Pelowski, 2014), even after controlling for stimulus-driven activity. For example, interaction-specific changes in coordination performance and EEG IBS have been reported for turn-based economic games (Astolfi *et al*., 2011; Jahng *et al*., 2017), turn-based verbal exchange (Ahn *et al*., 2018), turn-based target selection (Balconi *et al*., 2018), turn-based button pressing (Shiraishi and Shimada, 2021) and a turn-based card game (Astolfi *et al*., 2010a). It is worth noting that all studies cited here have implemented controls to check whether changes in IBS were due to turn-based interactions or merely due to the shared environment with shared stimuli. Therefore, we reasoned that previous research provides enough evidence to conclude that IBS measured with EEG has the potential to reflect coordination during turn-based interactions. While our turn-taking task affords a high degree of deliberation in order to infer mental states underlying a co-actor’s (predicted) behaviors (similar to previous single-participant ToM studies), it should be noted that this approach differs from more continuous tasks such as joint music making and joint body movement where such effortful deliberation on mental states underlying previous action outcomes is unlikely to be as relevant.

A caveat is that one may question whether ToM is necessary to perform this coordination task. If participants view it as a pattern recognition task, they may match responses to image feedback without necessarily conceptualizing the feedback as originating from another person’s mental states, thereby ostensibly bypassing the need for ToM processing. However, we feel this is unlikely for three reasons. First, participants are introduced to each other before beginning the experiment and are therefore primed to infer that image selection feedback originates from the person whom they met. Second, while it is possible for one participant in a dyad to adopt a leader strategy, selecting a decision rule on each trial without using ToM and expecting the other participant to follow, it is unlikely that participants would do this across trials since a single decision rule selected by the leader will not be applicable to many trials. This is because the color or shape upon which a single decision rule is based will not be present in the vast majority of trials. Therefore, during early stages of the task, participants frequently encounter trials where a previously established focal point is not present, giving both participants many opportunities to deviate from pure leader or follower roles. Third, they are explicitly told that, since they cannot verbally communicate, they should pay attention to what the other person chooses, and should remember that the other person is also paying attention to what they choose. Thus, participants are told the task involves a two-way cooperative effort.

It should be noted that the extent to which working memory affects two-person coordination is likely to depend on the type of coordination task. In the present study, working memory appears to be important at least for two-person tasks where participant performance depends on tracking previous actions. Not all forms of coordination require active, dynamic interactions where previous actions inform subsequent actions. One such example was given earlier, from Mehta *et al*. (1994) where a large proportion of participants gave ‘John’ as a response when asked to provide a name that a randomly chosen individual would also provide. The precise role of working memory in the emergence of focal points remains an important matter for further research. Based on previous findings from single-participant studies, working memory may play a role in both tracking the perspective of another person and in top-down selection between the perspective of oneself or another person (Qureshi and Monk, 2018). Working memory is also likely to be important for the ability to condition behavior on previous social outcomes (Duffy and Smith, 2014). In future work testing a causal role of working memory, two-person dual-task paradigms may be effective to extend single-participant findings which have correlated working memory in other types of synchronization, for example sensorimotor synchronization to fluctuating rhythms (Colley *et al*., 2018).

Overall, our findings reveal a causal role for working memory in the ability of individuals to use focal points, specifically during dynamic interactions where previous actions influence subsequent actions. Importantly, we also find that P3 is a meaningful marker for coordination performance in this context of dynamic interaction. This study demonstrates that, as the multi-agent approach to social neuroscience continues to develop, experimental manipulation and assessment of domain-general cognitive processes and related ERPs across interacting individuals may prove to be useful in testing new hypotheses for mechanisms underlying social coordination.

## Supplementary Material

nsae017_Supp

## Data Availability

The data and code underlying this article are available in Unishare at https://unishare.nl/index.php/s/HQZdbYQtDCdkr2y.
