# Supplementary figures and images for: Working memory load impairs tacit coordination but not inter-brain EEG synchronization
Source: Soc Cogn Affect Neurosci. 2024 Feb 28;19(1):nsae017. doi: 10.1093/scan/nsae017 (PMC10919395; doi:10.1093/scan/nsae017)

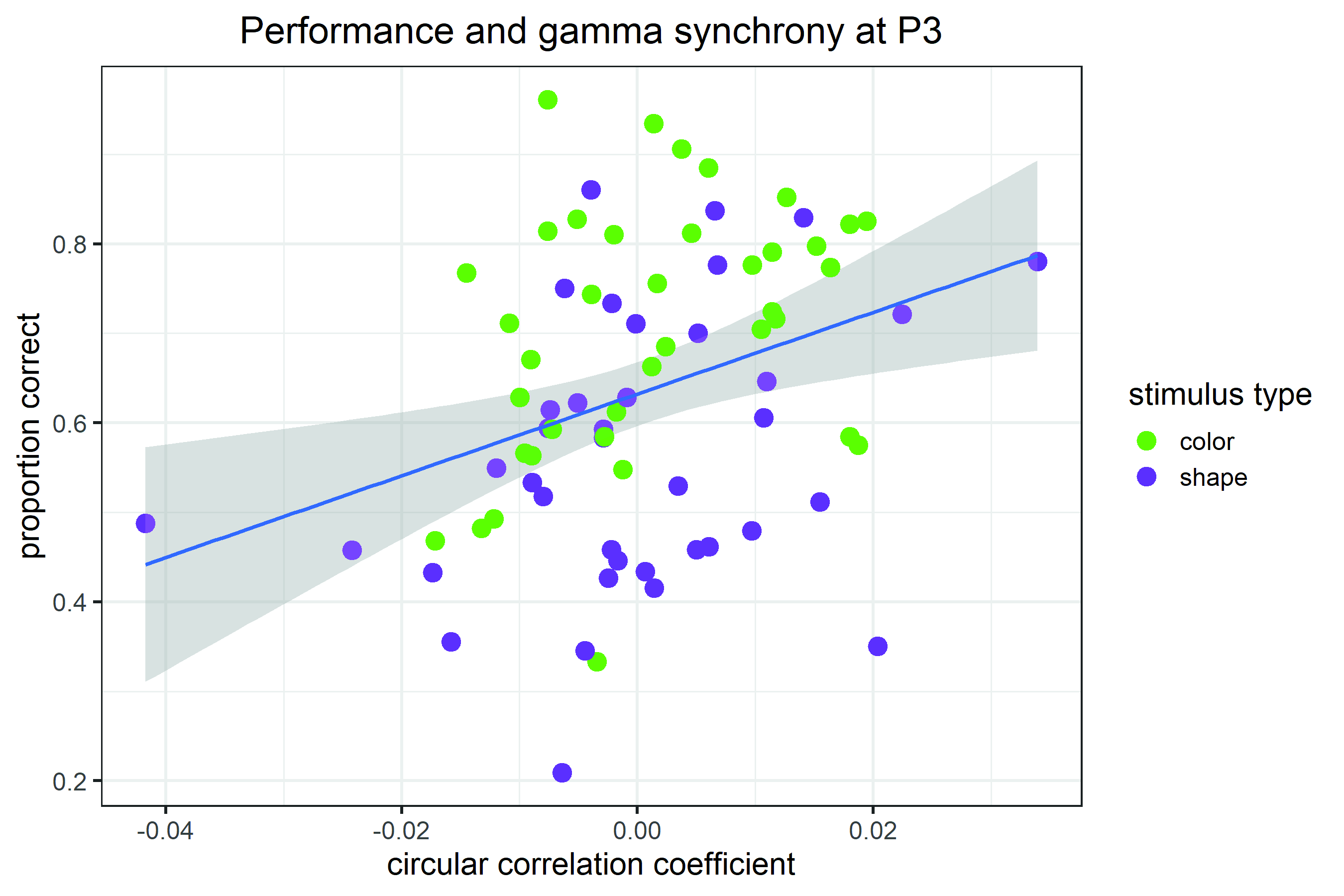

Supplement: nsae017_Supp [file nsae017_supp.zip › scan-22-202-File007.tif]
