# Supplementary material for: Working memory load impairs tacit coordination but not inter-brain EEG synchronization
Source: Soc Cogn Affect Neurosci. 2024 Feb 28;19(1):nsae017. doi: 10.1093/scan/nsae017 (PMC10919395; doi:10.1093/scan/nsae017)
Supplement: nsae017_Supp [file nsae017_supp.zip › scan-22-202-File002.doc]

**SUPPLEMENTARY MATERIAL**

**Methods**

*Questionnaires*

Prior to EEG setup and the computerized task, each participant completed questionnaires assessing social attitudes, skills, and traits. The Autism-Spectrum Quotient (AQ; Baron-Cohen et al., 2001) measures the degree to which adults with normal intelligence possess traits associated with the autism spectrum. The AQ measures five subscales, with higher scores indicating more neurodivergence in each area: social skills, attention switching, attention to detail, communication, and imagination. The Interpersonal Reactivity Index (IRI; Davis, 1983) measures four dimensions of empathy: perspective taking, fantasy, empathic concern, and personal distress. The Interaction Anxiousness Scale (IAS; Leary, 1983) is a measure for traits of social anxiety and was included because it has been previously found to significantly correlate negatively with behavioral coordination in a joint finger tapping task (Yu*n et a*l., 2012).

*Task*

In each of the four images displayed per trial, three colors or shapes alternated in a grid pattern, with two of the colors or shapes fixed across the four images in the trial, and one color or shape varying between the four images (Figure 1). The position of the varying color or shape in the alternating grid was randomized between trials. Presentation order of image sets (i.e., trials) was randomized for each dyad. Stimuli were generated such that each color and shape was presented an equal number of times in an equal number of trials, and no combination of three colors or shapes was repeated. This meant that participants could not make decisions based solely on memory for whether an image was previously chosen by their partner, but decisions instead required representations of image features and, ostensibly, the emergence of focal points corresponding to these feature representations.

For each n-back trial, there was a 33% chance of a 2-back match. No lures were given (i.e., the number at the 1-back position never matched the number at the 2-back position) in order to avoid imposing at random an additional load on working memory (Szmale*c et a*l., 2011). In both high and low load, participants were given 2.5 seconds to respond while the number was displayed, after which feedback was given. In the case of a correct response, green text displayed the word “CORRECT”; for an incorrect response, red text displayed “INCORRECT”; and if no response was given within the allotted time, red text displayed “TOO SLOW”. Mean accuracy on the number task in the current block was displayed. After this feedback was shown for 1 second, a jittered fixation cross was displayed for 1-3 seconds (to prevent EEG synchronization driven by task timing) before images were displayed and the image coordination phase of the trial began as described above.

At the beginning of each block, participants completed practice trials. In the high load block, before completing any image trials, they were first given five practice trials of a 1-back task, followed by five practice trials of a 2-back task. In the low load condition, they completed three practice trials of even-odd discrimination. Participants then completed two practice trials of the image coordination task, in which images were composed of shapes or colors different from those included in the main experimental trials. Afterwards, three final practice trials were given, combining both the number and image tasks in a single trial, again using shapes or colors different from those in the main experiment.

Participants were compensated with a base rate of €8 per hour (typically €16 for completing a two-hour session), plus a bonus of up to €4 more depending on their performance in both the image coordination task and the n-back task. They were instructed that accuracy in the number and image tasks both matter equally for the compensation bonus, and not to focus on one task more than the other. This was done in part to minimize the likelihood that participants would bypass the working memory load manipulation by focusing disproportionately on the image task while neglecting the n-back task. After the experiment ended, a score was calculated for each participant using the following formula:

0.5 * (0.66 * mean first-best guess accuracy + 0.33 * mean second-best guess accuracy) + 0.5 * mean n-back accuracy

Participants with a score >= 0.9 received a bonus of €4, those with a score < 0.9 and >=0.8 received €3, those with a score < 0.8 and >= 0.7 received €2, etc., with scores below 0.6 receiving no bonus.

*EEG Recording and Preprocessing*

In the BioSemi ActiveTwo daisychain mode, data from the “slave” AD-box is fed to the “master” AD-box via a fiber optic cable, from which it is sent to the USB receiver that sends all data and trigger information to the acquisition computer (see Figure 8 in Barraza et al., 2019). Electrodes were placed in the international 10-20 system and impedances were kept below 30 kΩ. Four external electrooculogram (EOG) electrodes were used to detect eye movement: two vertical (below and above left eye) and two horizontal (adjacent to lateral canthi of both eyes). Reference electrodes were placed on mastoids.

Offline pre-processing was performed with Fieldtrip open source software (Oostenveld, Fries, Maris, & Schoffelen, 2011) in MATLAB (version 2018b, MathWorks). Data was first re-referenced to the average of the mastoid electrodes separately for each participant and filtered with a band pass ranging from 0.1 to 50 Hz, with 60 seconds of mirror padding to prevent edge artifacts. For event-related potential (ERP) analyses, epochs were segmented starting at image onset and ending 1.5 seconds after image onset, linear trends were removed via detrending, and a baseline for each trial was removed from each channel as determined by the average of 1 second preceding stimulus onset. Trials were then rejected by visual inspection based on the presence of non-stereotyped artifacts (e.g., muscle activity and electrical noise) occurring over multiple channels. In trials with non-stereotyped artifacts occurred over a small number of channels, individual channels were interpolated for those trials only. For IBS analyses, epochs were segmented starting at the beginning of the feedback phase, after both participants had selected an image, and lasting 3 seconds. Trials with high-amplitude noise across channels (i.e., muscle activity) were then detected for rejection by first running a padded bandpass filter at 110-140 Hz where muscle activity typically occurs, then using the “ft_artifact_zvalue” function in FieldTrip to calculate the average z-normalized signal across channels at every time-point. Trials with z-values above and below a user-set threshold were visually inspected for each dyad to ensure the threshold captured artifacts and not signal; trials with z-values above the threshold were rejected. For both ERP and IBS analyses, channels with artifacts occurring in numerous trials across the session were interpolated across all trials. Then an Independent Component Analysis (ICA) was performed and components containing localized, high-powered noise, eye movements, or eye blinks were removed.

*Data Analysis*

*Linear Mixed-Effects Models.* Like many other studies in the cognitive sciences (Meteyard and Davies, 2020), we opted to use linear mixed-effects models (LMMs) and a model selection procedure rather than more traditional methods (like repeated measures analysis of variance) because LMMs maximize statistical power (due in part to their ability to account for dependence between samples while also allowing for random effects such as variability at the level of subjects), and simulations indicate that model selection with LMMs minimizes Type I error rate (Matuschek et al., 2017). LMMs were implemented in R (version 3.5.2; R Core Team, 2021) using the “lmer” function from the “lme4” package.

*Nonlinear changes in time-series.* GAMs work by optimally fitting the data with splines, which are functions determined as the weighted sum of any number of base functions, each of which has its own shape that can be linear or nonlinear. As the number of base functions increases, more complex relationships are captured by the model. The GAM can therefore be described as a further generalization of typical regression methods: while each variable has an intercept and a coefficient in a standard regression, in GAMs there is an intercept and the possibility for multiple coefficients, each representing the contribution of a base function. The model implements a penalized regression similar to that used in a standard regression, in which a penalty is associated with each coefficient to prevent over-fitting. To balance over- and under-fitting, a smoothing parameter is optimized when fitting base functions to the data, in our case using maximum likelihood (as with LMMs, GAMs that differ in fixed effects can only be compared if they were fit using maximum likelihood).

Model comparisons for GAMs were performed using the “compareML” function of the “mgcv” package in R. It compares two models on the basis of the minimized smoothing parameter selection score (maximum likelihood in our case), specifically by performing a chi-squared test on the difference in scores and the difference in degrees of freedom. The test calculates degrees of freedom by accounting for the number of estimated smoothing parameters, number of parametric (non-smooth) terms, and the sum of the penalty null space dimensions for each smooth object. It is therefore the preferred method for model comparison over other procedures such as AIC, specifically for models that include nonlinear random smooth effects (Wood, 2006; Wood, 2017).

*Questionnaires.* To test for a relationship between coordination performance and questionnaire scores, for each of the three questionnaires, a Pearson correlation was performed between accuracy in the coordination task (i.e., proportion of trials with matching responses) and questionnaire score (for total score and also for each subscale score).

*Event-related potentials.* ERPs are the result of averaging the EEG signal from many trials, time-locked to a specific stimulus. The P3 component in ERPs is a positive deflection in voltage in parietal areas, typically peaking around 250-500 ms after stimulus onset, and classically associated with selective encoding of task-relevant information to working memory (Donchin, 1981; Donchin and Coles, 1988). The presence of the P3 is thought to reflect updating in working memory for stimulus evaluation and decision-making, while its amplitude indicates demands on attentional resources (Sirevaa*g et a*l., 1989; Kramer and Spinks, 1991).

**Results**

*Questionnaires*

For each of the three questionnaires assessing social attitudes, skills, and traits, an LMM was fitted to test the effect of questionnaire score on coordination performance. For all three questionnaires, no significant effect was found (p>0.05).

| **Model number** | **Description** | **Outcome variable** | **Fixed effects** | **Random effects** |
| --- | --- | --- | --- | --- |
| 1 | LMM: Effect of WM load on mean coordination performance | mean accuracy for each dyad | 1. *WM load* 2. stimulus type | dyad  (random intercept) |
| 2 | GAM: Effect of WM load on change in performance over trials | trial-by-trial accuracy | 1. *WM load* (parametric) 2. stimulus type (parametric) 3. interaction between trial and load condition (spline) 4. interaction between trial and stimulus type (spline) | dyad (random factor spline) |
| 3 | LMM: Effect of WM load on mean P3 amplitude | mean P3 amplitude for each subject | 1. *WM load condition* 2. stimulus type | subject nested within dyad (random intercept) |
| 4 | LMM: Effect of P3 amplitude on coordination performance | mean accuracy for each dyad | 1. *dyad-level mean P3* | dyad (random intercept) |
| 5 | LMM: Effect of WM load on mean IBS | mean IBS for each dyad | 1. *WM load*   2. stimulus type | dyad (random intercept) |
| 6 | LMM: Effect of IBS on mean coordination performance | mean accuracy for each dyad | 1. *IBS*  2. stimulus type | dyad (random intercept) |

**Supplementary Table 1. Model details**. Italics indicate variables of interest which were included in full models but excluded in reduced models for model selection. LMM = linear mixed-effects model; GAM = generalized additive model; WM = working memory; IBS = inter-brain synchronization.

**Supplementary Table 2. Summary statistics for model 1 (LMM for effect of load on accuracy).** Model results for the full LMM (including both load condition and stimulus type; model 1) and a reduced model (excluding load condition) are shown. Significant effects of load condition and stimulus type on mean accuracy were found. Chi-square test and AIC revealed a preference for the full model compared with the reduced model (χ2(1)= 3.90, p=0.048; ΔAIC=1.90). Bold p-values indicate statistical significance (α = 0.05).

**Supplementary Table 3. Summary statistics of model 2 (GAM for effect of load on change in performance over trials).** Load condition significantly affected (nonlinear) changes in accuracy over trials, as represented by a significant interaction between load condition and trial (F=4.71, edf=1.004, p=0.031). Smooth terms are indicated by “s()”.

**Supplementary Figure 1. Relationship between performance and gamma synchronization at the P3 electrode.** Points represent mean accuracy and mean IBS in gamma band for each dyad in each condition (two points per dyad, one for each block, i.e. condition). Blue line indicates regression line, gray shaded area indicates 95% confidence intervals. After testing for strength of evidence in favor of the null hypothesis, Bayes Factor analysis revealed strong evidence in favor of an effect of IBS on accuracy (BF_01_=0.077) in one out of 160 analyses (32 electrodes * 5 frequency bands): at the P3 electrode in the gamma band. In initial model comparisons (model 6), no effect of IBS on accuracy was found after correcting for multiple comparisons with FDR.

**REFERENCES**

Baron-Cohen, S., Wheelwright, S., Skinner, R., et al. (2001). The Autism-Spectrum Quotient (AQ): Evidence from Asperger Syndrome/High-Functioning Autism, Males and Females, Scientists and Mathematicians. *Journal of Autism and Developmental Disorders*, **31**, 5–17

Barraza, P., Dumas, G., Liu, H., et al. (2019). Implementing EEG hyperscanning setups. *MethodsX*, **6**, 428–36

Davis, M.H. (1983). Measuring individual differences in empathy: Evidence for a multidimensional approach. *Journal of Personality and Social Psychology*, **44**, 113–26

Donchin, E. (1981). Surprise!...surprise? *Psychophysiology*, **18**, 493–513

Donchin, E., Coles, M.G.H. (1988). Is the P300 component a manifestation of context updating? *Behavioral and Brain Sciences*, **11**, 357

Kramer, A., Spinks, J. (1991). *Capacity views of human information processing.* John Wiley & Sons.

Leary, M.R. (1983). Social Anxiousness: The Construct and Its Measurement. *Journal of Personality Assessment*, **47**, 66–75

MathWorks MATLAB and Statistics Toolbox Release 2012b

Oostenveld, R., Fries, P., Maris, E., et al. (2011). FieldTrip: Open source software for advanced analysis of MEG, EEG, and invasive electrophysiological data. *Computational Intelligence and Neuroscience*, **2011**

Sirevaag, E., Kramer, A.F., Coles, M.G.H., et al. (1989). Resource reciprocity: An event-related brain potentials analysis. *Acta Psychologica*, **70**, 77–97

Wood, S.N. (2017). *Generalized Additive Models: An Introduction with R, Second Edition*. 2nd Editio. Boca Raton: Chapman and Hall/CRC.

Wood, S.N. (2006). *Generalized additive models: An introduction with R*. Boca Raton, Florida, U. S. A.: Chapman & Hall.

Yun, K., Watanabe, K., Shimojo, S. (2012). Interpersonal body and neural synchronization as a marker of implicit social interaction. *Scientific Reports*, **2**, 959
